# Emerging Albumin-Binding Anticancer Drugs for Tumor-Targeted Drug Delivery: Current Understandings and Clinical Translation

**DOI:** 10.3390/pharmaceutics14040728

**Published:** 2022-03-28

**Authors:** Hanhee Cho, Seong Ik Jeon, Cheol-Hee Ahn, Man Kyu Shim, Kwangmeyung Kim

**Affiliations:** 1 Department of Materials Science and Engineering, Seoul National University, Seoul 08826, Korea; ricky@kist.re.kr (H.C.); chahn@snu.ac.kr (C.-H.A.); 2 Biomedical Research Institute, Korea Institute of Science and Technology (KIST), Seoul 02792, Korea; d21627@kist.re.kr; 3 KU-KIST Graduate School of Converging Science and Technology, Korea University, Seoul 02841, Korea

**Keywords:** albumin, drug delivery system, cancer-targeted therapy, prodrug

## Abstract

Albumin has shown remarkable promise as a natural drug carrier by improving pharmacokinetic (PK) profiles of anticancer drugs for tumor-targeted delivery. The exogenous or endogenous albumin enhances the circulatory half-lives of anticancer drugs and passively target the tumors by the enhanced permeability and retention (EPR) effect. Thus, the albumin-based drug delivery leads to a potent antitumor efficacy in various preclinical models, and several candidates have been evaluated clinically. The most successful example is Abraxane, an exogenous human serum albumin (HSA)-bound paclitaxel formulation approved by the FDA and used to treat locally advanced or metastatic tumors. However, additional clinical translation of exogenous albumin formulations has not been approved to date because of their unexpectedly low delivery efficiency, which can increase the risk of systemic toxicity. To overcome these limitations, several prodrugs binding endogenous albumin covalently have been investigated owing to distinct advantages for a safe and more effective drug delivery. In this review, we give account of the different albumin-based drug delivery systems, from laboratory investigations to clinical applications, and their potential challenges, and the outlook for clinical translation is discussed. In addition, recent advances and progress of albumin-binding drugs to move more closely to the clinical settings are outlined.

## 1. Introduction

The targeted drug delivery systems have emerged to achieve an efficient delivery of anticancer drugs to the tumors [1]. In particular, various nanomaterials, such as polymeric nanoparticles, dendrimers, micelles, liposomes and inorganic nanoparticles involve tumor-targeted drug delivery and have thus received considerable attention over the past decades [2,3,4,5,6,7]. These nanomaterials allow a reduced drug dosage for minimal side effects while resulting in more efficient drug dosing to the tumors to achieve the desired therapeutic efficacy by passive and active targeting [8,9,10,11]. Passive targeting describes the phenomenon where nanomaterials can be extravasated from the leaky angiogenic vessels and efficiently accumulated in the tumor tissues via the enhanced permeability and retention (EPR) effect [12,13,14]. Furthermore, nanomaterials have great amenability to modify with ligands, including peptides, small organic molecules, oligosaccharides and antibodies for targeting tumor cells by active targeting [15]. With these distinct targeting mechanisms, nanomaterials have constituted a central system for targeted drug delivery, but only a few candidates are clinically approved. This is because of several intrinsic shortcomings of the systems, i.e., (i) potential toxicity of nanomaterials and (ii) their fairly complex structure and synthetic processes, hindering quality control (QC) and scale-up industrial production. Therefore, there is a desperate need for safe and more precise targeted drug delivery systems for effective cancer treatment [16].

**Figure 1:**
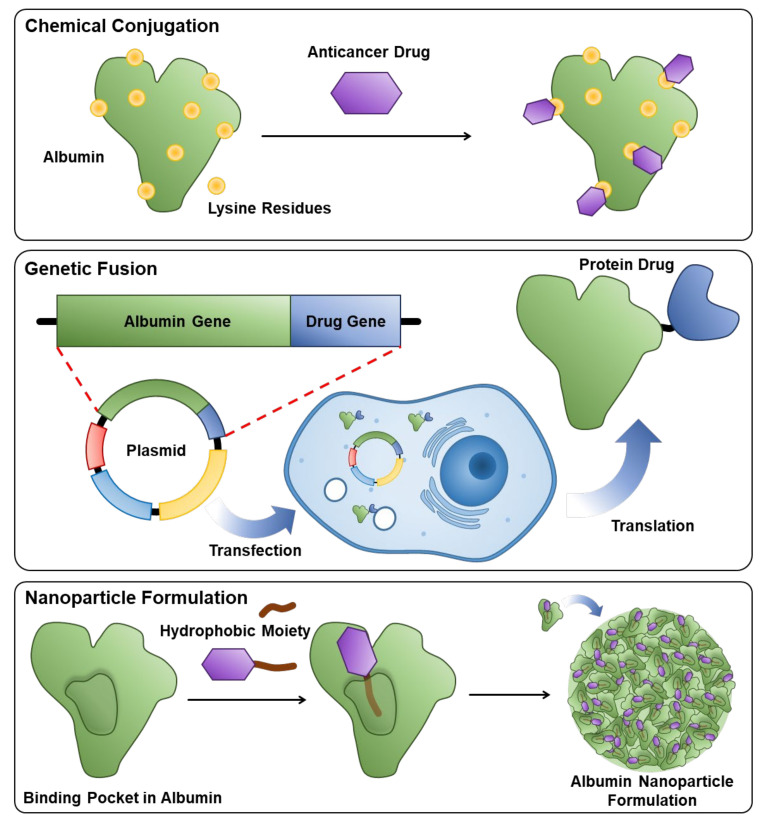
Schematic illustrations to explain three methods for the preparation of exogenous albumin-bound drug formulation.

Albumin, an important nutrient source for the body, has been extensively explored as a natural drug delivery system due to its several advantages, such as high biocompatibility, easy surface modification and good biodegradability (Figure 1) [17]. Importantly, albumin can be inherently formulated in the hydrophobic anticancer drugs in its hydrophobic interiors, thereby improving pharmacokinetic (PK) profiles with an enhanced circulatory half-life of drugs for tumor-targeted delivery [18,19]. Its long circulation is predominately attributable to the neonatal Fc receptor (FcRn)-mediated cellular recycling and Megalin/Cubilin complex that rescues albumin from renal clearance [20,21]. In addition, albumin-bound drug formulations efficiently accumulate within tumors by the EPR effect that is shown in the macromolecular complex by the increased vascular permeability and low lymphatic drainage [22,23]. Accordingly, several candidates have been evaluated clinically, and the most successful example is Abraxane, an exogenous human serum albumin (HSA)-bound paclitaxel formulation approved by the FDA in 2005 [24]. However, the additional clinical translation of exogenous albumin formulations has not been approved to date because many anticancer drugs cannot be easily formulated with albumin owing to their intrinsic planar structure, polarity and crystallization tendency [25]. Most importantly, even an effective drug delivery system has been analyzed to have unexpectedly low delivery efficiency, with less than 1% of administered nanoparticles reaching the tumors in many preclinical studies [26,27]. It means that a considerable amount of drugs is accidently released at off-target sites, which can increase the risk of systemic toxicity.

**Figure 2:**
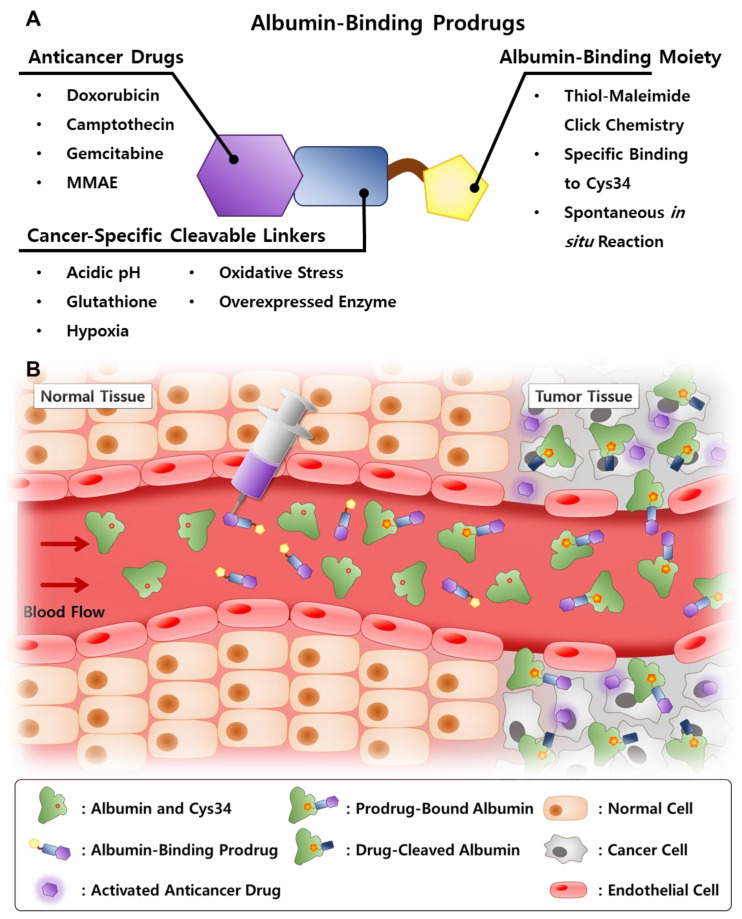
(**A**) Structure of the albumin-binding prodrug and explanations about each constituent. (**B**) Schematic illustration of the mechanism of action of albumin-binding prodrug.

To overcome these obstacles in the clinical translation of the albumin-based drug delivery system, anticancer drugs have been chemically conjugated with albumin-binding moieties that covalently bind to the endogenous albumin, which allows the application of more various types of drugs without consideration for their intrinsic characteristics (Figure 2) [28]. Their simple and accurate structure is easy for a scale-up industrial production with precise quality control (QC), and the covalent binding of albumin and anticancer drugs prevents drug leakage during circulation. In particular, the critical design is the cancer-specific cleavable linkers between albumin-binding moieties and anticancer drugs to release the payload selectively in the tumors. Selective drug release can be achieved via the design of prodrugs by exploiting intrinsic differences between the tumor and normal tissues; in particular, hypoxia, low pH and diverse enzymes overexpressed in tumors are promising biomarkers for targeted drug delivery [29]. As a result, these prodrugs can bind covalently to in situ circulating albumin for targeting tumors via passive accumulation and selectively release the anticancer drugs in the tumors owing to structural cleavage by biomarkers, while systemic toxicity is reduced by minimal drug release in normal tissues [30,31,32,33,34]. The first and most advanced prototype of the albumin-binding prodrug is the (6-maleimidocaproyl)hydrazone derivative of doxorubicin, Aldoxorubicin, which is constructed with albumin-binding maleimide molecule, a pH-sensitive linker of hydrazone and doxorubicin, which is now in phase III clinical trials [35]. Encouraged by these active clinical approaches, many designed albumin-binding prodrugs that can selectively release the drugs by different biomarkers in tumors have been developed. In this review, we give the account of the albumin-based drug delivery from different aspects, including preparation and properties, half-life extension, targeting mechanisms and therapeutic applications. In addition, past exogenous albumin-bound anticancer drug formulations are summarized from laboratory investigations to clinical applications, and their potential challenges and the outlooks for clinical translation are discussed. Finally, recent advances and progress of endogenous albumin-binding prodrugs to move more closely to the clinical settings are outlined.

## 2. Properties of Albumin

### 2.1. Human Serum Albumin (HSA)

HSA is the most abundant protein in blood plasma, presenting in human serum at a concentration of 33–52 g/L [36]. It is mainly synthesized by hepatocytes at a rate of 0.2 g/kg per day and immediately secreted to blood after its production. HSA not only circulates within blood vessels, but about 60% of its total mass in the human body is located in extravascular tissue. Intravascular and extravascular HSA are continuously exchanged with each other at a rate of 4–5%/h through the neonatal Fc receptor (FcRn)-mediated transcytosis mechanism, and extravascular HSA is excreted through the lymphatic system to rejoin the bloodstream [37]. HSA is finally catabolized inside various organs, and the degraded albumin can be a nutrient to tissues [38]. Approximately 3.7% of intravascular albumin is secreted or degraded and freshly synthesized at the same rate every day [20]. The blood half-life of albumin is ~20 d.

The primary structure of HSA is a single polypeptide composed of 585 amino acid sequences, which has 66.5 kDa molecular mass and ~10 nm hydrodynamic diameter [39,40]. It contains 16.8% of acidic amino acid and 16.9% of basic amino acids, and its isoelectric point is 4.7 [41,42]. HSA has 67% of α-helices but no β-sheet as its secondary structure [43]. Its tertiary structure is found to be heart shaped with three main domains (I to III), each consisting of two helical subdomains (A and B) [44]. There are 35 cysteine residues forming 17 disulfide bonds themselves, and only one unpaired thiol presents on subdomain IA [45]. HSA is highly water soluble, since it has lots of basic or acidic residues, and its sufficient amount of disulfide groups leads to its high stability at adverse pH or temperature conditions [46]. Thanks to its abundance in blood plasma and multi-functionality in its structure, albumin plays an important role in the homeostasis of the body, such as pH and colloid osmosis [47]. Its complex structure also allows it to construct both ionic and hydrophobic binding sites inside and transport a variety of nutrients and biomolecules, such as hormones, lipids and amino acids [48].

### 2.2. Other Types of Albumins

As an alternative to HSA, albumins from a variety of other sources, such as bovine serum albumin (BSA), rat serum albumin and ovalbumin (OVA), are commercially available at a much lower cost. OVA is a single-chain phosphoglycoprotein derived from the egg white. With globular 3D structures, it has 45 kDa molecular mass and ~3 nm hydrodynamic diameter [49,50]. OVA is composed of 386 amino acid sequences, including six cysteines, and two of them are coupled with a disulfide bond. Due to its pH and temperature sensitivity, OVA is highly applicable for a stimuli-sensitive drug delivery system [51,52,53]. The secondary and tertiary structures of OVA, however, are far different from those of HSA. It has four unpaired thiol groups, whereas the HSA holds only one, and its hydrophobic binding pocket is not similar to that of HSA, making it difficult to be used for the preparation of albumin-binding anticancer drugs.

BSA is a single-chain polypeptide with 583 amino acid residues, with a molecular mass of 66.8 kDa and an isoelectric point of 4.7 [54]. Including the heart-shaped tertiary structure, three domains consisting of six subdomains and a single unpaired thiol group, the structure of BSA is 76% homologous to that of HSA, since both are mammalian albumins [55,56,57]. This structural similarity facilitates the use of BSA as a powerful alternative to HSA, exploiting the advantage of its low cost and negligible immunogenicity as well [58]. Albumins obtained from other mammalian species, such as rats, rabbits and pigs, are commercialized as well; however, only a few studies using them have been reported yet.

## 3. Albumin Binding of Anticancer Drugs

### 3.1. Albumin-Binding Methods

Since albumin has a number of free functional residues and their secondary structures, different types of anticancer drugs can be bound to it in a variety of ways. By binding drugs to albumin, their pharmacokinetic properties can be improved, showing better biodistribution and bioavailability. Binding methods can be generally classified into two categories, non-covalent and covalent manners. The non-covalent method utilizes the secondary structures of albumin where drug molecules are reversibly bound through ionic or hydrophobic interactions. There are two main binding pockets in its substructures, which are named Sudlow site I and II [59]. Sudlow site I, also known as warfarin-azapropazone site or fatty-acid-binding site (FA) 7, is located in subdomain IIA and was originally intended for the transportation of native fatty acids and lipids [60,61,62]. Sudlow site I binds certain types of hydrophobic drugs, such as derivatives of warfarin, azapropazone and phenylbutazone [63,64,65]. Sudlow site II, also called indole-benzodiazepine site or FA3-FA4, is in subdomain IIIA and can stereo-selectively interact with drugs, such as profen, naproxen and diazepam [18,66,67,68]. Hydrophobic interaction, as well as hydrogen bond and ionic interaction, are involved in drug formulation in Sudlow site II [69,70]. In addition to Sudlow site I and II, a binding site in subdomain IB, analogous to FA1, has been recently reported as a secondary pocket for several drugs, such as indomethacin, naproxen, warfarin and bilirubin [71,72]. There are other binding sites called FA2, 5, 6, 8 and 9, which bind fatty acids via simple hydrophobic interactions [73,74,75].

As albumin contains lots of residues, including lysine, aspartic acid, cysteine and serine, drugs can be attached with covalent bonding. Amines in albumin can react with drug molecules through N-hydroxysuccinimide-activated ester, isocyanate or aldehyde chemistry, and carboxylic acids or hydroxyl groups are also able to be exploited for drug conjugation. These chemical modifications, however, are rarely used for the preparation of albumin-binding anticancer drugs, since they cannot specify the binding site of drugs and lack reproducibility. These approaches even cause the denaturation of albumin and impact its circulation and metabolism. In this circumstance, the use of thiol chemistry with the 34th cysteine residue (cys34) is of the greatest interest for anticancer drug conjugation [28,46]. Cys34 is the sole unpaired thiol in the native albumin and is located on its surface. The simple thiol-maleimide (Thiol-Mal) click chemistry enables the drug–albumin conjugation with high site specificity and reproducibility without compromising the basic structure of albumin. The thiol-Mal click chemistry is especially applicable for in situ drug binding, as the thiol present in albumin accounts for 80% of the thiol content in the total blood plasma [76].

Genetic fusion of protein/peptide drugs with albumin is another covalent binding method. By fusing the human albumin expressing gene and protein/peptide drug encoding gene, active proteins/peptides are selectively introduced to the N-terminal of recombinant albumin without impairing its structure. The fusion of albumin and protein drugs is notably advantageous for protein-based therapeutics, since most protein drugs have high immunogenicity, short half-life and low bioavailability [28]. A variety of protein drugs, including cytokines, enzymes, growth factors and hormones, have been successfully fused with albumin throughout the previous studies, exhibiting enhanced pharmacokinetics and therapeutic efficacy [77,78,79,80,81]. The types of drugs that can be fused with recombinant albumin via this technology are limited to protein/peptide-based ones. Moreover, the covalent binding methods, including both chemical modification and genetic fusion, have a problem in that the drug must be dissociated from albumin in the final step to recover its active form. The linker between the drug and albumin should be cleavable under the tumor microenvironment or drugs that exhibit their efficacy regardless of albumin binding should be chosen.

### 3.2. Albumin Binding and Anticancer Drug Half-Life

Conventional anticancer drugs have suffered from low therapeutic efficacy due to various factors, one of which is their poor biodistribution and, thereby, rapid elimination from the body. The conjugation of poly(ethylene glycol)s (PEGs) or albumin to drug molecules are two popular strategies widely used elsewhere to improve their pharmacokinetic profiles [82,83]. Non-specific PEGylation of drugs, however, may hinder their approach to the site of action, resulting in deterioration of their activities. The non-biodegradability and slight immunogenicity of PEG are further drawbacks compared to albumin [84,85]. Albumin binding can be a better candidate than PEGylation for the drug half-life extension with its non-immunogenicity and biodegradability. For example, P. Kurtzhals et al. conjugated hydrophobic fatty acid chain to the LysB29 residue of human insulin analog [86]. As the fatty acid on the insulin tightly binds to albumin, the half-life of insulin (4–6 min) was increased by more than 50 times (5–7 h). This insulin analog finally obtained FDA approval for treatment of type 1 or 2 diabetes and has been available on the market as Levemir^®^ since 2005 [87,88].

There are some albumin receptor-mediated pathways that elongate the half-life of albumin despite its biodegradability. These receptors allow albumin to be recycled and transported without undergoing lysosomal degradation [18,20]. The glycoprotein (GP) 60 receptor, also known as albondin, is an albumin receptor present restrictively on the membrane of vascular continuous endothelium cells and alveolar epithelial cells [18,89]. GP60 binds native albumin and provokes the association of caveolin-1, forming albumin-containing vesicles named caveolae. The caveolae move into the cytoplasm, fuse with the basal membrane and finally transport albumin to the interstitium [90]. The transcytosis of albumin by the GP60 receptor rescues it from lysosomal degradation and extends its half-life. FcRn is another cell membrane receptor involved in the transcytosis of albumin. Different from GP60, FcRn can be broadly found in various cell membranes, such as vascular endothelium, intestinal epithelium, interstitium of the central nervous system, renal epithelium, etc. [91]. Native albumin in the physiological condition generally has a low affinity for FcRn. As it undergoes endocytosis, albumin strongly interacts with FcRn, specifically under the endosomal pH (6–6.5). The FcRn-bound albumin can effectively evade lysosomal degradation and be released back to the interstitium [92,93]. It has been reported that FcRn plays a major role in maintaining the albumin concentration, as a significant decrease in albumin was observed from the FcRn-deficient mice [94,95].

Contrary to the action of GP60 and FcRn, GP18 and GP30 facilitate the removal of denatured or modified albumin [96]. Predominantly expressed on the membrane of hepatic endothelium and peritoneal macrophage, GP18 and GP30 bind abnormal albumin and induce its degradation via caveolae-related endocytosis [97,98,99,100]. Albumin with denatured conformation binds to those receptors with a 1000-fold higher affinity than the native one. As seen in the G18- and GP30-mediated pathways, it is certain that excessive chemical modification of albumin with drugs may lead to its conformational change, which is disadvantageous to the pharmacokinetics of drug–albumin conjugate. In addition, Megalin/Cubilin receptor is usually located on the membrane of kidney proximal tubule cells, preventing the renal excretion of albumin [101,102]. Megalin and Cubilin form a complex with each other, and the albumin binds to Megalin to be endocytosed. K. Weyer et al. demonstrated that the Megalin/Cubilin complex is essential for inhibiting renal clearance of albumin using a Megalin/Cubilin-deficient mouse model [103]. However, the interaction of albumin with Megalin/Cubilin receptor does not extend the lifespan of albumin-binding anticancer drugs, since the albumin translocated into cells via Megalin/Cubilin receptor-mediated endocytosis is instantly degraded by lysosomes [104].

### 3.3. Albumin Binding and Cancer Targeting

Since it was revealed that tumor tissues extensively uptake plasma proteins, albumin has been intensively studied as a carrier for various anticancer drugs, such as doxorubicin, paclitaxel, gemcitabine, monomethyl auristatin E (MMAE) and camptothecin, due to its abundance in serum [105,106]. There are two fundamental mechanisms for the tumor accumulation of albumin: the EPR effect and receptor-mediated tumor internalization and catabolism [107,108,109]. In normal tissues, macromolecules, including albumin, generally cannot pass through the densely connected vascular endothelium but are only transported via transcytotic mechanisms. The migrated macromolecules are smoothly excreted from tissues through the lymphatic system. On the contrary, solid tumors are often hypervascularized with abnormally organized leaky blood vessels and a lack of lymphatic vasculature. Due to the highly permeable architecture of tumoral blood vessels, macromolecules are freely extravasated into tumor interstitium, hardly excreted by lymphatic drainage, and thereby accumulated in the tumor tissue [110,111,112]. This phenomenon is widely known as the EPR effect and has been actively explored for the passive targeting of cancer with macromolecular and nanoparticulated therapeutics.

After its passive accumulation in tumor tissues, albumin is captured by overexpressed receptors and selectively endocytosed to cancer cells. Secreted protein acidic and rich in cysteine (SPARC), also known as osteonectin or basement membrane 40 (BM40), is the central receptor contributing to the entrapment and catabolism of albumin inside the cancer cell [90]. The expression of SPARC is strictly regulated in normal cells, whereas it is excessively present on cancer membranes [113,114]. When the SPARC receptor recognizes and binds to albumin, the complex is endocytosed to cancer cells and then undergoes degradation. The interaction of SPARC with native albumin is considered to be similar to that of GP60; however, its intuitive experimental proof has not been performed yet [89,115]. In contrast to the EPR effect, SPARC-related spontaneous uptake by cancer cells occurs selectively for the albumin. Attributed to these passive and active tumor-targeting mechanisms, albumin has been considered a versatile cargo for anticancer agents.

## 4. Exogenous Albumin-Bound Anticancer Drug Formulations for Cancer Therapy

### 4.1. Developments in Exogenous Albumin-Bound Anticancer Drug Formulations

As the usefulness of albumin in terms of its pharmacokinetic property and tumor specificity was demonstrated, considerable attempts have been made to develop albumin-based anticancer drug carriers. Stehle et al. synthesized methotrexate (MTX)–albumin conjugates for anticancer therapy [116,117]. MTX, which has a short half-life of 6–10 h, was covalently bound to lysine residues in albumin at a molar ratio of 1:1. The pharmacokinetics of MTX were notably improved, exhibiting 15–18 d of half-life, which is similar to that of native albumin. After confirming its antitumor efficacy in animal models, this MTX–albumin conjugate was named MTX-HSA and entered clinical trials in the early 2000s [118]. There are some structural problems with MTX-HSA due to its synthetic method. It is hard to regulate the specific site of MTX conjugation in the albumin structure, and MTX is rarely released unless the albumin is degraded. Recently, a couple of studies were carried out for the synthesis of exogenous albumin-bound anticancer drugs using the Cys34 as the conjugation site of drugs [119,120]. Utilizing the thiol-Mal click chemistry, the site-specificity-related issues present in MTX-HSA could be partially cleared.

For the conjugation of protein/peptide-based drugs to albumins, genetic fusion is a practical approach widely adopted throughout numerous research works. Among albumin-binding protein drugs, antibody-bound albumins are frequently used for cancer treatment. P. J. Yazaki et al. manufactured a fusion protein with albumin and single-chain antibody (scFv) of carcinoembryonic antigen (CEA) to target human colorectal carcinoma [121]. The fusion of albumin to anti-CEA successfully complemented the rapid clearance of anti-CEA, demonstrating a dramatic increase in its tumor-specific accumulation up to 4.6-fold. Since the fusion of protein drugs to albumins rarely affects the structure of albumin, other hydrophobic anticancer drugs can be additionally loaded to the binding pockets of albumin [122]. D. Dong et al. fused the antibody of the human epidermal growth factor receptor 2 (anti-HER2) to albumin and loaded the fatty-acid-conjugated fluorescein isothiocyanate (FA-FITC) to it as a model drug [123]. The FA-FITC was stably bound to albumin-fused anti-HER2 via hydrophobic interaction, and the albumin-fused anti-HER2 was efficiently uptaken by HER2-overexpressing cancer cells. In another study, multiple proteins were fused together with albumin [124]. The antibodies of vascular endothelial growth factor A (anti-VEGF-A) and hepatocyte growth factor (anti-HGF) were fused to construct a multi-domain protein. The fusion of multiple antibodies to albumin enabled to overcome the resistance of cancer against single antibody, as well as improving their pharmacokinetics. This multi-domain fusion protein was named M0250 and entered the clinical evaluation.

### 4.2. Methods for Albumin Nanoparticle Formation

Strategies to prepare albumin nanoparticles have already been fully established throughout decades of research. These strategies can be classified as either chemical or physical methods, depending on how albumins aggregate themselves and what the driving force is that maintains the particle morphology. Desolvation and emulsification methods belong to the former, while nanoparticle-albumin-bound (NAB) technology, thermal gelation, nanospray drying, self-assembly and protein salting out are the latter. Each technique for albumin nanoparticle formation has its distinguished pros and cons, so it should be carefully selected according to the desired particle property and the type of drug to be loaded.

Desolvation, initially designed by C. Weber et al., is one of the widely used methods for albumin nanoparticle formation [125]. This method utilizes the difference in solubility of albumin to water and ethanol. When ethanol is dropwisely added to the aqueous albumin solution with continuous stirring, the albumin is gradually precipitated as a nanoparticle due to the decrease in its solubility to the co-solvent [126]. The precipitated nanoparticle is then chemically cross-linked utilizing difunctional aldehyde linkers, amination between albumin residues or disulfide exchange reactions for its further stabilization [127,128,129]. The desolvation method enables obtaining highly stable albumin nanoparticles with freely adjustable sizes according to pH and temperature changes. During this particle formation procedure, however, albumin would be denatured due to the addition of ethanol or changes in pH and temperature [130,131].

The emulsification method exploits the phase separation between oil and water. An aqueous albumin solution is poured into an excessive amount of oil phases, such as cotton seed or castor oil, forming droplets of the albumin solution with rapid agitation. After heating the emulsion to evaporate water, the albumin nanoparticles dispersed in the oil phase are chemically crosslinked, mixed with an organic solvent, such as ether or cyclohexane, and centrifuged to remove the viscous oil [132,133,134]. Similar to the desolvation method, particle size can be easily controlled via the emulsification method, but denaturation of albumin is inevitable. The potential toxicity from the residual organic solvent also hinders its clinical application [135].

NAB technology is developed by American Bioscience, Inc. for the encapsulation of hydrophobic drugs into albumin nanoparticles [24]. This technique is well known for the production method of Abraxane^®^ (paclitaxel-albumin nanoparticle), which is the sole albumin-bound paclitaxel nanoformulation approved by the FDA [136]. The organic solution of hydrophobic drugs and the aqueous albumin solution are mixed for their complexation, and the mixture is homogenized under high pressure. The tertiary structure of drug-bound albumin is partially unfolded by mechanical shear stress, and the unfolded albumin is aggregated into nanocrystals through electrostatic and hydrophobic interactions [137,138]. The NAB technology is exclusively beneficial due to its high productivity, but the anticancer drugs that can be loaded in albumin nanoparticles through this method are limited to hydrophobic ones. Several drugs developed with the NAB technology are currently undergoing clinical trials [139].

The mechanism of thermal gelation resembles that of NAB technology. In the procedure of thermal gelation, deformation of the albumin structure occurs, followed by reassembly into nanoparticles via hydrogen bonding, electrostatic and hydrophobic interactions [140]. Thermal gelation can be accompanied by the intermolecular disulfide exchange reaction, resulting in the chemical crosslinking of albumin [141]. Unfortunately, there are no studies that have formed nanoparticles with albumin alone, and mixing with other macromolecules such as chitosan, dextran and lysozyme, is necessary for it [142,143].

Spray drying is an industrial method to produce ultra-fine powder formulations for nasal and pulmonary drug delivery [144]. For the formation of albumin nanoparticles through nanospray drying, albumin and drugs are dissolved together, and the solution is sprayed through a small-bore nozzle to make nanosized aerosols. Albumin solution aerosol is dried under reduced pressure by heating, and the resulting nanopowder is collected using an electrostatic precipitator [145,146]. The solution that contains albumin and drug must be extremely clear; otherwise, the nozzle may be clogged. Nanospray drying is a promising technique for the manufacture of albumin nanoparticles with its simple procedure and high capability of mass production.

The self-assembly technique exploits the hydrophobic interaction of chemically modified albumins for their nanoformulation. As the native albumin is highly water soluble, moieties, such as hydrophobic polymers, drugs or fatty acids, should be conjugated to modulate its property to be amphiphilic. Poly(l-lactic acid), doxorubicin and fatty acids with various carbon lengths have been studied as hydrophobic moieties for self-assembled albumin nanoparticles [147,148,149]. The chemical conjugation of hydrophobic moieties consumes the lysine residues in the albumin structure, further reducing the hydrophilicity of albumin itself.

The protein salting-out method is a relatively recently introduced technique for the fabrication of albumin nanoparticles, which induces an ionic intermolecular interaction between albumins by adding ionic salts. Mixing the aqueous albumin solution and ionic crosslinking agents in ethanol solution by their comparable volume ratios, the albumin is precipitated by the change in its solubility and physically crosslinked by ionic salts at the same time [150,151]. This technique is quite similar to the desolvation method, except for not using a chemical crosslinker. Sodium phosphate or potassium phosphate is mainly used as an ionic crosslinking agent.

The several inorganic nanoparticles can be applied to develop the albumin nanoparticles [152]. The nanosized inorganic materials, such as gold and iron oxide, are highly unstable in normal condition and carry the risk of potential toxicity; thus, their surface modification is widely accepted to improve in vivo fate and allow the conversion into promising nanoparticles [153]. Therefore, many researchers developed inorganic nanoparticle-based albumin nanoparticles by coating nanoparticles with albumin and using them as drug carriers to improve the properties of anticancer drugs [154]. Kim et al. modified the surface of gold nanoparticles with albumin via redox-liable disulfide linkage [155]. Consistently, the albumin-modified gold nanoparticles showed significantly enhanced antitumor efficacy with high tumor accumulation. Nosrati et al. coated the iron oxide nanoparticles with albumin via the surface charge adsorption method. The albumin formulation of iron oxide nanoparticles improved colloidal stability and biocompatibility for magnetic hyperthermia therapy [156].

### 4.3. Developments in Albumin-Bound Anticancer Drug Nanoformulations

According to the methods for drug binding and albumin nanoparticle formation, various types of albumin nanoparticles have been investigated to improve the therapeutic effect of anticancer drugs and minimize their side effects. X. Yu et al. chemically modified gemcitabine with myristic acids (Gem-C14s) and encapsulated them in albumin nanoparticles [157]. Gem-C14s interacted with the hydrophobic binding sites of albumins, and Gem-C14-bound albumins were formulated via NAB technology. The size of the albumin nanoparticle was measured to be 150 ± 27 nm, and the nanoparticle encapsulated Gem-C14 with an efficiency of 82.99 ± 3.5%. In another study, docetaxel-bound albumin nanoparticles (DTX-NPs) were formulated through the protein salting out [158]. Docetaxel was loaded to albumin nanoparticles via non-specific hydrophobic interaction, and its loading efficiency was up to 63.1%. The size of DTX-NPs was controlled to less than 200 nm. DTX-NPs exerted lower systemic toxicity than free docetaxel while maintaining their anticancer activity.

In addition to anticancer drugs, other types of therapeutic substances can be loaded into albumin nanoparticles. H. Jeong et al. prepared photosensitizer-conjugated HSA nanoparticles for the tumor photodynamic therapy [159]. Chlorine e6 (Ce6), a photosensitizer that has three carboxylic acid groups in its molecular structure, was mixed with HSA and chemically crosslinked together through the amination to yield Ce6-HSA nanoparticles with a size of 88 nm. The nanoformulation of Ce6 with HSA successfully improved the tumor-specific biodistribution of hydrophobic Ce6. Similarly, IR780 iodide-bound albumin nanoparticle was developed for its application in photodynamic and photothermal dual therapy [160]. IR780 was bound to denatured albumin via hydrophobic interaction; then, the denatured albumin spontaneously aggregated itself to form particles of ~250 nm in diameter. IR780 acted as a photosensitizer and a photothermal agent depending on the wavelength of the irradiated laser. The use of albumin nanoparticles as siRNA carriers can enhance their transfection efficiency. S. Son et al. synthesized siRNA-bound HSA nanoparticles by crosslinking the siRNA and HSA with disulfide bonds [161]. The siRNA was significantly stabilized within the nanoparticle, and its accumulation to the tumor was increased by 1.7 times.

Albumin nanoparticles are utilized for the co-delivery of therapeutic agents to promote their synergistic anticancer efficacy. B. Kim et al. fabricated the paclitaxel and curcumin co-bound albumin nanoparticles (PTX/CCM Alb-NPs) for the treatment of pancreatic cancer [162]. As both paclitaxel and curcumin are hydrophobic, they were identically bound to albumins, and the complexes were formulated through NAB technology. PTX/CCM Alb-NPs improved the pharmacokinetic properties of paclitaxel and curcumin and exhibited a 71% higher anticancer effect compared to PTX Alb-NPs. Other combinations of anticancer drugs, including paclitaxel and pirarubicin, paclitaxel and all-trans-retinoic acid, or docetaxel and gemcitabine, have been examined for their co-binding to albumin nanoparticles and application in cooperative cancer therapy [157,163,164]. X. Yu et al. incorporated photosensitizers rather than anticancer drugs to gemcitabine-bound albumin nanoparticles for the combined chemotherapy and photodynamic therapy of pancreatic cancer [165]. Pheophorbide was chemically conjugated to albumin as a photosensitizer. After binding Gem-C14 to albumin, the particle was formulated via NAB technology. The pheophorbide in the nanoparticle not only produced reactive oxygen species as a photosensitizer but also assisted the imaging of the tumor site as a fluorescence dye. Likewise, the co-binding of drugs and imaging agents, such as gold nanocluster and fluorescence dye, to albumin nanoparticles leads to the production of theranostic nanomedicines [21,166].

Albumin nanoparticles possess abundant functional residues on their surfaces, so that various moieties endowing supplementary functionalities can be introduced to them. S. Choi et al. modified the surface of doxorubicin-conjugated albumin nanoparticles with tumor-necrosis-factor-related apoptosis-inducing ligands (TRAILs) [167]. Doxorubicin and octyl aldehyde were primarily conjugated to albumins to yield self-assembled nanoparticles, and TRAILs were chemically linked on their surfaces. TRAILs on the nanoparticle surface boosted the apoptosis of cancer cells collaboratively with doxorubicin. In another study, folic acids were conjugated on the surface of gemcitabine-loaded albumin nanoparticles (FA-Gem-BSANPs) as targeting moieties [168]. Gem-BSANPs were formulated through the desolvation method, and folic acids were introduced via amination reaction. Folic acids on the surface of the nanoparticle facilitated its intracellular uptake by folate receptor-overexpressing tumors. Biotin (biotin-specific receptor), trastuzumab (HER2 targeting antibody) and CREKA (tumor homing peptide) have also been assessed to provide albumin nanoparticles with additional active targeting of tumors [169,170,171]. The cationic polymers, such as polyethyleneimine or poly(l-Lysine), are another candidate for the surface modification of albumin nanoparticles. It was confirmed that the coating of the albumin nanoparticle surface with cationic polymers enhances its cellular internalization [172].

### 4.4. Clinical Translation of Exogenous Albumin-Bound Anticancer Drug Formulations

Based on the good therapeutic outcomes and progressive achievements in a series of research works, several exogenous albumin-bound anticancer drug formulations are commercially available or in clinical trials. The related clinical trials are listed in Table 1.

Among them, MTX-HSA is the first formulation that had undergone clinical evaluations [28]. The phase I study showed that MTX-HSA has a maximum tolerated dose (MTD) of 50 mg/m^2^ for every 2 weeks, and its major dose-limiting toxicity was stomatitis [173]. Its phase II trial was conducted on patients with renal cell carcinoma; however, no therapeutic response was observed [174]. After the phase II trial, MTX-HSA has been tried in combination with other anticancer drugs against various types of cancers, but it does not seem to be progressing anymore.

Some recombinant albumins have also been in clinical trials, including MM-111 and M0250, which have been designed for cancer therapy. MM-111, developed by Merrimack Pharmaceuticals, Inc., is recombinant albumin fused with the bispecific scFv for HER2/HER3 receptors (anti-HER2/HER3) [175,176]. Albumin-fused anti-HER2/HER3 efficiently prolonged their in vivo circulation with half-life of 16–20 h, which is 3–4 times longer than that of free antibody [177]. Since its phase I/II study is still ongoing, there are few reports about the therapeutic results of MM-111. M0250 is a recombinant albumin-based drug delivery system that has most recently started clinical trials [124]. It was designed by Molecular Partners AG and entered phase I/II trials in 2014. In the phase I test, M0250 exhibited its half-life of 15 d and MTD of 8 mg/kg every 2 weeks. Its dose-limiting toxicities were thrombotic microangiopathy, gastrointestinal hemorrhage, nephrotic syndrome, etc. [178,179].

Abraxane^®^, also known as nanoparticle albumin-bound paclitaxel (nab-paclitaxel), was developed by Abraxis BioScience and obtained FDA approval in 2005 for the treatment of metastatic breast cancer, locally advanced or metastatic non-small cell lung cancer and metastatic adenocarcinoma of the pancreas [137]. Paclitaxel is hardly soluble in water and has been conventionally used by mixing with Cremophor EL^®^ into a formulation, called Taxol^®^ [180]. However, the Cremophor EL^®^ in Taxol^®^ was revealed to induce side effects, such as hypersensitivity reaction, hyperlipidemia and peripheral neuropathy [181]. Abraxane^®^ was initially designed to manufacture formulations with improved pharmacokinetic properties without using Cremophor EL^®^ and has been rapidly replacing Taxol^®^. Produced by NAB technology, Abraxane^®^ has a particle size of ~130 nm, and the ratio of paclitaxel to albumin is 1:9 [182]. As it is injected intravenously, Abraxane^®^ selectively accumulates via GP60- and SPARC-mediated pathways [183]. The terminal half-life and AUC of Abraxane^®^ were determined to be 27 h and 18,741 ng/mL, respectively [184]. In phase I studies, Abraxane^®^ showed LD50 of 47 mg/kg and MTD of 300 mg/m^2^ for every 3 weeks with sensory neuropathy, stomatitis and ocular toxicity as dose-limiting toxicities, whereas Taxol^®^ had LD50 value of 30 mg/kg and 175 mg/m^2^ [185,186]. Its phase II studies were carried out in patients with metastatic breast carcinoma using an Abraxane^®^ dose of 300 mg/m^2^ per 3 weeks, demonstrating 48% of overall response rate with remarkably relieved paclitaxel-associated adverse effects [187]. Its anticancer activity was finally compared to paclitaxel solution in phase III, approving its superiority with 33% of overall response rate against metastatic breast cancers (19% for paclitaxel solution) [188]. Abraxane^®^ also exhibited notable therapeutic outcomes in combination with other agents, including trastuzumab, bevacizumab, carboplatin, 5-fluorouracil or gemcitabine [189,190,191,192,193].

Encouraged by clinical advances of Abraxane^®^, anticancer drugs other than paclitaxel, such as docetaxel, rapamycin and analogs of thiocolchicine, are also bound to albumin nanoparticles through the same method and have entered clinical evaluations [194,195,196]. All of these albumin nanoparticles are found to have significantly reduced toxicity and improved pharmacokinetics compared to the free forms of anticancer drugs, which is similar to the clinical results of Abraxane^®^.

Despite the successful commercialization of some drug formulations, the exogenous albumin-binding anticancer drugs have limitations that diminish their therapeutic efficacy. Firstly, recent findings noted that the delivery efficiency of nanoparticles is desperately low, i.e., no matter how effective the delivery system is, its amount to reach the tumor cannot exceed 1% of the total injection. The remaining 99% of drug delivery systems are misled to off-target sites and cause undesired systemic toxicity by premature drug leakage, which is recognized as a chronic complication of nanoparticle-based drug carriers. The reproducibility- and quality-control-related issues in nanoparticle formation are another hurdle for the use of exogenous albumin-binding anticancer drug formulations. Furthermore, the use of exogenous albumin is possibly accompanied by the risk of its deterioration or contamination, and the denaturation of albumin during the nanoparticle formation process is assumed to shorten its half-life [28]. For example, Abraxane^®^ obviously circulates in the blood much longer than free paclitaxel or Taxol^®^, but its half-life is only 5% of that of native albumin. Since all preparation methods for albumin nanoformulation inevitably bring about the denaturation of albumin, the problem associated with the reduction in half-life is very hard to solve. In addition, many anticancer drugs of great hydrophobicity are exclusively hard to formulate with albumins, and high-yield synthesis of albumin is not a trivial task [197]. In these circumstances, endogenous albumin-binding drugs can be better candidates for cancer treatment.

## 5. Endogenous Albumin-Binding Anticancer Drugs for Cancer Therapy

Compared to exogenous albumin-bound anticancer drug formulation approaches, anticancer drugs modified with albumin-binding moieties that can covalently conjugate with albumin can bind endogenous albumin for targeted drug delivery after intravenous administration [198]. These drug conjugates have a relatively precise and concise structure to achieve industrial scale-up production with appropriate quality control (QC), and most anticancer drugs can be adopted without consideration of their intrinsic characteristics [199]. In particular, introducing cancer-specific cleavable linkers between drug and albumin-binding moiety allows selective drug release in tumors. Selective drug release can be achieved via the design of prodrugs for targeting heterogeneous features in tumors, such as morphology, biology and biochemistry; in particular, the tumor microenvironment is reflected in the various biomarkers, such as hypoxia, low pH and diverse range of overexpressed enzymes compared to normal tissues [200]. As a result, the underlying mechanism is that these prodrugs can bind covalently to endogenous circulating albumin to target tumors and selectively release the anticancer drugs owing to structural cleavage by biomarkers, while systemic toxicity is reduced by minimal drug release in normal tissues. With these distinct advantages, the first and most advanced prototype of the albumin-binding prodrug is Aldoxorubicin (formerly known as INNO-266), which consist of albumin-binding maleimide moiety, a pH-sensitive linker of hydrazone, and doxorubicin [201]. In phase II clinical trials, Aldoxorubicin significantly improved the median survival of leiomyosarcoma patients, and the overall tumor response rate by independent review was higher with Aldoxorubicin than with doxorubicin [35]. In addition, Aldoxorubicin showed superior progression-free survival (PES) in a relapsed or refractory settings of advanced soft tissue sarcomas compared to the free DOX in phase III clinical trials [202]. As summarized in Table 2, encouraged by successful clinical studies, many researchers have developed albumin-binding prodrugs that can selectively release the anticancer drugs by different biomarkers in tumors, with enhanced tumor accumulation via the high affinity to the endogenous albumins.

The basis of the progression- and transformation-related biochemical imbalance in tumors results in a difference in the amount, expression pattern and activity of certain enzymes [203]. These enzymes play crucial roles in regulating and maintaining the tumor microenvironment that consists of tumor cells, abnormal vasculature and extracellular matrix (ECM) [204]. As a result, tumor cells in the tumor microenvironment overexpress diverse types of enzymes compared to normal cells [205]. Enzymes in the tumor cells are unique biomarkers for the diagnosis and treatment, especially cathepsins, caspases, matrix metalloproteinases (MMPs) and other enzymes that have proteolytic activities to recognize and cleave specific peptide sequences in proteins, which are pivotal in many mechanisms in cancer biology [206,207,208]. Thus, many researchers have developed albumin-binding prodrugs that contain peptide substrates specifically cleaved by enzymes in tumors.

**Figure 3:**
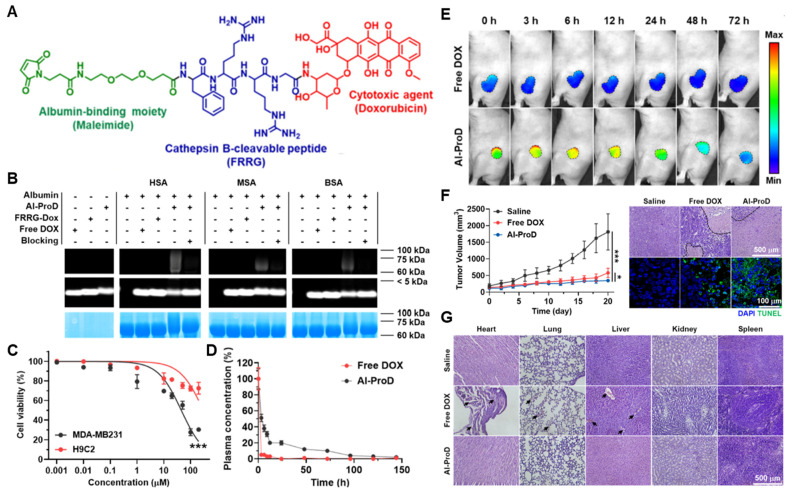
**Cathepsin B-specific albumin-binding prodrug.** (**A**) Albumin-binding maleimide moiety is conjugated with doxorubicin through cathepsin B-specific cleavable peptide FRRG. (**B**) The resulting Al-ProD is efficiently bound to three types of albumin (HSA, MSA and BSA). (**C**) Al-ProD induces a potent cytotoxicity in MDA-MB231 breast cancer cells, whereas cytotoxicity is greatly minimized in H9C2 cardiomyocytes. (**D**) Al-ProD shows extended half-life in vivo via binding with endogenous albumins compared to free DOX. (**E**) With extended half-life, Al-ProD efficiently accumulates within tumor tissues. (**F**) Al-ProD significantly delays tumor progression by inducing a potent apoptosis in tumor tissues. (**G**) Al-ProD greatly minimizes the systemic toxicity by selective drug release in tumor tissues. * *p* < 0.05 and *** *p* < 0.001. Reproduced with permission from [216]. Copyright 2022, Cho, et al.

Cathepsin B, a lysosomal cysteine protease, plays a prominent role in intra- and extracellular proteolysis, which is one of the 11 human cysteine cathepsins [209,210]. Many clinical results have demonstrated that cathepsin B is involved in the degradation of the ECM for tumor progression and metastasis and is thus overexpressed in most malignancies [211,212]. Accordingly, several peptide substrates of cathepsin B have been exploited for tumor-targeted drug delivery, which showed promising outcomes for the treatment of tumors in multiple preclinical trials [213,214,215]. As an application of cathepsin B substrate peptide for the design of albumin-binding prodrugs, Cho et al. developed cathepsin B-overexpressed tumor-cell-activatable albumin-binding doxorubicin prodrug (Al-ProD), constructed with albumin-binding maleimide, cathepsin B-specific cleavable peptide FRRG and doxorubicin (Figure 3A) [216]. In in vitro assays, the Al-ProD efficiently bound to three types of albumin (HSA, BSA, MSA) and induced 30-fold strong cytotoxicity in cathepsin B-overexpressed MDA-MB231 breast cancer cells compared to cathepsin B-deficient H9C2 cardiomyocytes (Figure 3B,C). After intravenous administration, Al-ProD showed a significantly extended in vivo half-life than free DOX via binding with endogenous albumin, thereby increasing tumor accumulation (Figure 3D,E). As a result, Al-ProD treatment resulted in distinctly delayed tumor progression with minimized side effects during treatment compared to free DOX (Figure 3F,G). As a similar approach, Kratz et al. proposed albumin-binding cathepsin B-specific prodrug (HSA-1), which consists of a maleimide group, cathepsin B-specific cleavable FL peptide, self-immolative linker (PABC) and doxorubicin [217]. The in vivo properties of HSA-1 were evaluated in MDA-MB435 breast tumor models, and the standard and maximum tolerated dose (MTD) was evaluated as 3 × 24 mg/kg, which is a good estimate of the MTD compared to doxorubicin (2 × 8 mg/kg). Importantly, free DOX exhibited low antitumor efficacy at its optimal dose of 2 × 8 mg/kg, while HSA-1-treated mice showed minimal tumor growth at the dose of 3 × 24 mg/kg.

**Figure 4:**
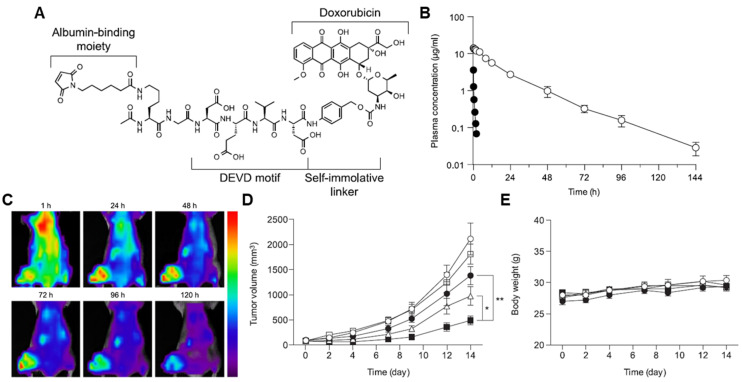
**Caspase-3-specific albumin-binding prodrug.** (**A**) Albumin-binding maleimide moiety is conjugated with doxorubicin through caspase-3-specific cleavable peptide KGDEVD and self-immolative linker (PABC), resulting in EMC-DEVD-S-DOX. (**B**) EMC-DEVD-S-DOX shows long half-life (~19 h). (**C**) EMC-DEVD-S-DOX efficiently accumulates within tumor tissues with detectable substances for 120 h. (**D**,**E**) EMC-DEVD-S-DOX significantly inhibits the tumor growth with minimal systemic toxicity. * *p* < 0.05 and ** *p* < 0.005. Reproduced with permission from [222]. Copyright 2016, Elsevier.

Caspases are an evolutionary conserved family of cysteine proteases that are involved in cell death [218]. Currently, at least 14 different caspases have been confirmed to exist, which cleave their substrates in the carboxyl group of the aspartate residue [219]. In particular, caspase-3 is the most well-known biomarker, as it plays a critical role in the intrinsic and extrinsic cell death receptor pathways [220]. The caspase-3-mediated tumor-targeted drug delivery systems are combined with other treatments, such as photodynamic therapy (PDT), photothermal therapy (PTT) and radiation (RT) [221,222,223,224,225]. This is because apoptosis should be preceded for overexpression and activation of caspase-3 to trigger the subsequent release of anticancer drugs from the systems. Chung et al. developed an albumin-binding caspase-3-specific cleavable prodrug (EMC-DEVD-S-DOX; MPD01), constructed with albumin-binding moiety maleimide, caspase-3-cleavable peptide KGDEVD, self-immolative spacer (PABC) and doxorubicin (Figure 4A) [222]. The EMC-DEVD-S-DOX showed a significantly extended terminal half-life of more than 19 h, which was approximately 40-fold longer than that of DEVD-S-DOX (albumin non-binding prodrug; Figure 4B). With extended in vivo circulation, EMC-DEVD-S-DOX bound to endogenous albumin efficiently accumulated within the tumor tissues with a detectable amount of the substances for more than 120 h (Figure 4C). After tumor accumulation, a tumor volume was decreased by 99% compared to the control group when combined with local RT for caspase-3 activation to trigger subsequent doxorubicin release, and noticeable body weight changes during treatment did not occur (Figure 4D). This group also proposed an albumin-binding caspase-3-specific cleavable monomethyl auristatin E (MMAE) prodrug (MPD02) [226]. The MPD02, consisting of an albumin-binding maleimide group, caspase-3-specific cleavable peptide KGDEVD, self-immolative spacer and MMAE, greatly minimized the systemic toxicity of free MMAE with no noticeable reduction in the body weight during treatment. In triple-negative breast cancer xenograft models, the mice treated with MPD02 with local RT showed a high tumor growth inhibitor (TGI) of 99% compared to the saline group. The clinical use of MMAE is strictly hindered owing to its severe toxicity despite its 100–1000 times stronger efficacy than doxorubicin, whereas MPD02 efficiently reduced MMAE-related side effects [227].

MMPs are a family of extracellular enzymes involved in the degradation of ECM for tumor progression, metastasis and recurrence [228]. Normally, MMPs are regulated in an inactive state by tissue inhibitors of metalloproteinases, hormones, growth factors and cytokines [229]. However, the activation and overexpression of MMPs are observed in advanced tumors; in particular, the activity and expression levels of MMPs are significantly upregulated in late-stage and malignant tumors [230]. Therefore, MMPs are valuable biomarkers to achieve tumor-targeted drug delivery [231]. Mansour et al. proposed an albumin-binding prodrug containing maleimide, MMP-2-specific cleavable peptide GPLGIAGQ and doxorubicin [232]. Against A375 melanoma that expressed high amounts of MMP-2, prodrugs exhibited distinctly superior efficacy to doxorubicin at an equivalent dose and minimized the severe lymphopenia that was observed in doxorubicin-treated mice.

Prostate-specific antigen (PSA) is a serine protease overexpressed in prostate carcinoma and a promising biomarker [233]. Drevs et al. developed PSA4, constructed with maleimide, PSA-specific peptide spacer (SSYYSG) and doxorubicin [234]. The PSA4 showed a 5–7-fold increase in the maximum tolerated dose (MTD) in the mice compared to doxorubicin and induced good antitumor activity in PSA-positive DuPro-1 tumor models. In addition, even 3-fold or more doses of PSA4 (24 mg/kg) did not induce body weight change, while doxorubicin (8 mg/kg) treatment resulted in severe systemic toxicity with significant body weight loss (−36%) compared to control group.

**Figure 5:**
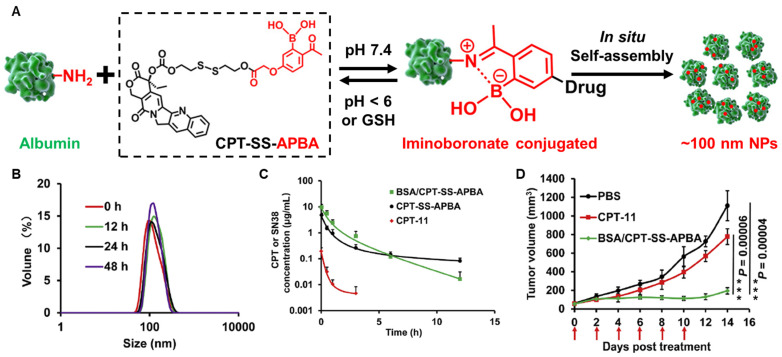
**GSH-specific albumin-binding prodrug.** (**A**) Albumin-binding 2-acetylphenylboronic acid (APBA) is conjugated with camptothecin (CPT) through GSH-specific cleavable S-S bond bound to albumin endogenously and self-assembled in situ to nanoparticles. (**B**) The resulting CPT-SS-APBA induces in situ self-assembly to form nanoparticles with improved stability in mouse serum. (**C**) CPT-SS-APBA extends half-life of free camptothecin via binding with in situ albumin. There were no significant differences in the plasma concentration profiles of CPT-SS-APBA and BSA/CPT-SS-APBA administered after binding with albumin in vitro. (**D**) CPT-SS-APBA shows a potent antitumor efficacy compared to free camptothecin. Reproduced with permission from [198]. Copyright 2022, American Chemical Society.

In addition to diverse enzyme biomarkers, oxidative stress in the tumor microenvironment by a high concentration of glutathione (GSH) and reactive oxygen species (ROS) can be target for efficient drug delivery [235]. In this oxidative condition, the concentrations of ROS (100–1000 × 10^−6^) and GSH (2–20 μM) in the tumors are 100-fold and 7–10-fold higher than those in normal tissues, respectively [236]. Therefore, oxidative stress in the tumors rather than other normal tissues is represented and can provide a potential strategy for the design of albumin-binding prodrugs. Sun et al. designed an albumin-binding gemcitabine prodrug that consists of maleimide, GSH-specific cleavable S-S linker and gemcitabine [237]. The albumin binding of the gemcitabine prodrugs was completed within 1 min, and albumin-bound gemcitabine prodrugs exhibited cytotoxicity specifically in oxidative stress in vitro. Because of the high hydrophilic structure, gemcitabine showed poor pharmacokinetic parameters in the mice, whereas gemcitabine prodrugs increased the area under the curves (AUC) via endogenous albumin binding approximately 9-fold. As a result, gemcitabine prodrugs efficiently accumulated in the tumor tissues and induced a potent antitumor efficacy compared to free gemcitabine. Hao et al. conjugated camptothecin (CPT) with the albumin-binding moiety of 2-acetylphenylboronic acid (APBA) via GSH-specific cleavable S-S bonds (Figure 5A) [198]. The resulting CPT-SS-APBA readily bound to albumin and induced in situ self-assembly to from nanoparticles with improved colloidal stability (Figure 5B). As a result, CPT-SS-APBA greatly improved the pharmacokinetic profiles with 4-fold higher AUC and longer half-life than free gemcitabine and significantly delayed the tumor progression compared to free camptothecin (*p* = 0.0006) and control (*p* = 0.00004) groups (Figure 5C).

**Figure 6:**
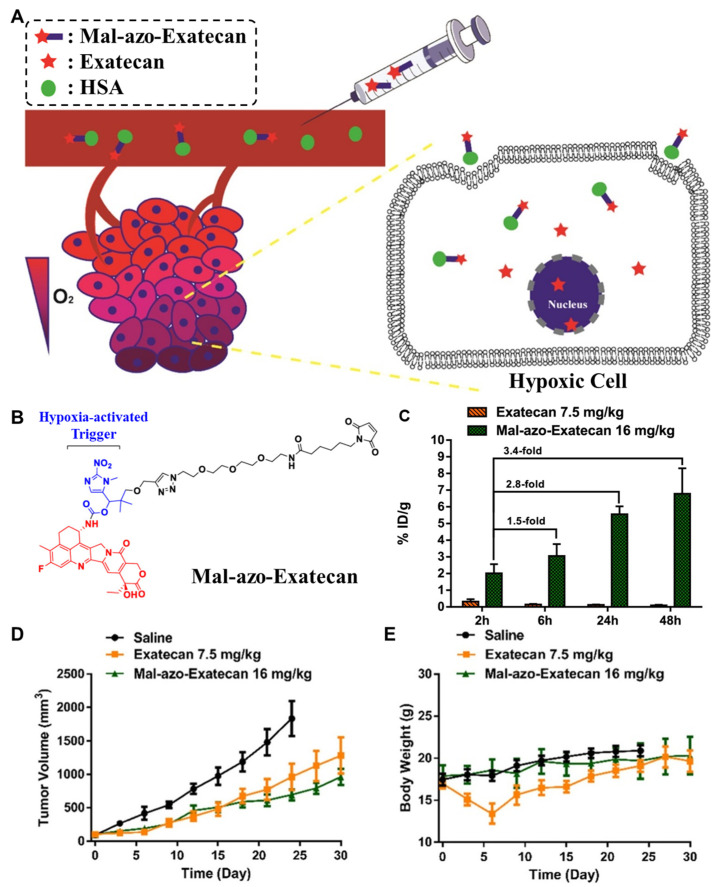
**Hypoxia-specific albumin-binding prodrug.** (**A**) Schematic illustration depicting the mechanism of action of hypoxia-specific albumin-binding prodrug. (**B**) Albumin-binding maleimide moiety is conjugated with exatecan through hypoxia-specific cleavable linker 2-nitroimidazole, resulting in Mal-azo-Exatecan. (**C**) Mal-azo-Exatecan showed higher tumor accumulation than free exatecan. (**D**) Mal-azo-Exatecan exhibited superior antitumor efficacy to free exatecan, (**E**) while minimizing the systemic toxicity. Reproduced with permission from [244]. Copyright 2022, American Chemical Society.

Tumor hypoxia is a fundamental feature found in most solid tumor microenvironments [238,239,240]. The oxygen levels in most solid tumors are much lower than those in normal tissues owing to their extensive metabolism [241]. Treating hypoxic tumors is one of the most important directions in cancer oncology, including hypoxia-targeted drugs that selectively release the anticancer drugs by hypoxia-inducible factors (HIFs) and their signaling pathways [242]. In addition, certain reductases are more highly overexpressed in tumors than in normal tissues, and thus a series of hypoxia-specific cleavable linkers have been studied [243]. By using this framework, Cheng et al. developed hypoxia-specific albumin-binding prodrugs (Mal-azo-Exatecan) [244] (Figure 6A). They conjugated the albumin-binding maleimide group with the camptothecin analog, exatecan, via the hypoxia-specific cleavable linker of 2-nitroimidazole (Figure 6B). The in vivo distribution was evaluated in H460 tumor-bearing mice. The results showed that free exatecan was mainly observed in the livers and spleens with low tumor accumulation, whereas Mal-azo-Exatecan accumulated within tumor tissues 3.4-fold highly via efficient in situ albumin-binding (Figure 6C). With a high hypoxic selectivity of Mal-azo-Exatecan, the tumor growth was significantly more minimized than control and free exatecan groups (Figure 6D). Finally, free exatecan group exhibited a 20% body weight loss at end of the treatment, while Mal-azo-Exatecan minimized systemic toxicity, and body weight changes were similar with control group (Figure 6E).

**Table 1:** Albumin-binding anticancer drugs in clinical trials.

Carrier Type	Trade Name	Therapeutic Agent	Target	Clinical Stage	Reference
Native albumin, exogenous	MTX-HSA	Methotrexate	Renal cell carcinoma, advanced or metastatic transitional cell carcinoma	Phase II	[174,245]
Recombinant albumin	MM-111	HER2/HER3 antibory (anti-HER2/HER3)	Breast neoplasm, Her2-amplified solid tumors, metastatic breast cancer	Phase I/II	[246,247] NCT01097460, NCT00911898
	M0250	Vascular endothelial growth fact of-A antibody (anti-VEGF-A), hepatocyte growth factor antibody (anti-HGF)	Advanced solid tumors	Phase I/II	[178,179] NCT02194426
Albumin nanoparticle	Abraxane^®^	Paclitaxel	Metastatic breast cancer, locally advanced or metastatic non-small cell lung cancer, Metastatic adenocarcinoma of the pancreas	Approved	[188] NCT01583426
	ABI-008	Docetaxel	Hormone-refractory prostate cancer	Phase I/II	NCT00477529
	ABI-009	Rapamycin	Non-muscle invasive bladder cancer, solid tumors, PEComa, metastatic colorectal cancer, high grade recurrent glioma and newly diagnosed glioblastoma, soft tissue sarcoma	Phase I/II	NCT02009332, NCT00635284, NCT02494570, NCT03439642, NCT03463265, NCT03660930
	ABI-010	17-Allylamino-17-demethoxygel danamycin	Solid tumors	Withdrawn (prior to Phase I)	NCT00820768
	ABI-011	Thiocolchicine dimer	Solid tumors and lymphoma	Phase I	NCT02582827
Native albumin, endogenous	Aldoxorubicin	Doxorubicin	Soft tissue sarcoma, glioblastoma, HIV positive Koposi’s sarcoma, pancreatic ductal adenocarcinoma	Phase III	[35] NCT02049905, NCT02014844, NCT02029430, NCT01580397

**Table 2:** Endogenous albumin-binding anticancer drugs.

Albumin-Binding Moiety	Cancer-Specific Cleavable Linker	Anticancer Drug	Reference
Maleimide	Cathepsin B-specific cleavable FRRG peptide	Doxorubicin	[216]
Maleimide	Cathepsin B-specific cleavable FL peptide	Doxorubicin	[217]
Maleimide	Caspase-3-specific cleavable KGDEVD peptide	Doxorubicin	[222]
Maleimide	Caspase-3-specific cleavable KGDEVD peptide	MMAE	[226]
Maleimide	MMP-specific cleavable GPLGIAGQ peptide	Doxorubicin	[232]
Maleimide	Prostate-specific antigen (PSA)-specific cleavable SSYYSG peptide	Doxorubicin	[234]
Maleimide	GSH-specific cleavable S-S linker	Gemcitabine	[237]
2-acetylphenylboronic acid (APBA)	GSH-specific cleavable S-S linker	Camptothecin	[198]
Maleimide	Hypoxia-specific cleavable azo linker	Exatecan	[244]

## 6. Conclusions

The research and clinical application for albumin-based drug delivery systems were summarized from the past to the present. In this review, we described the general properties of albumin for drug delivery. In addition, the development processes and clinical translation of exogenous albumin-bound anticancer drug formulations were discussed. Finally, recent progress of endogenous albumin-binding prodrugs was introduced in detail. The mass clinical results from multiple failure cases of past albumin-based drug delivery systems would give valuable comments to develop the advanced albumin-binding prodrugs of the future. Continuous efforts to understand recent trends and progress for albumin-based drug delivery systems will provide important insights into designing more suitable methods for their clinical translation.

## Data Availability

Not applicable.
